# Olive Leaf Extract (OLE) impaired vasopressin-induced aquaporin-2 trafficking through the activation of the calcium-sensing receptor

**DOI:** 10.1038/s41598-021-83850-5

**Published:** 2021-02-25

**Authors:** Marianna Ranieri, Annarita Di Mise, Mariangela Centrone, Mariagrazia D’Agostino, Stine Julie Tingskov, Maria Venneri, Tommaso Pellegrino, Graziana Difonzo, Francesco Caponio, Rikke Norregaard, Giovanna Valenti, Grazia Tamma

**Affiliations:** 1 Department of Bioscience, Biotechnology and Biopharmaceutics, University of Bari Aldo Moro, Via Orabona 4, 70125 Bari, Italy; 2 Department of Clinical Medicine, Aarhus University, Aarhus, Denmark; 3 Department of Soil, Plant and Food Sciences, University of Bari Aldo Moro, Bari, Italy

**Keywords:** Kidney, Cell signalling, Cellular imaging, Membrane trafficking, Post-translational modifications, Cell biology, Molecular biology, Physiology, Diseases

## Abstract

Vasopressin (AVP) increases water permeability in the renal collecting duct through the regulation of aquaporin-2 (AQP2) trafficking. Several disorders, including hypertension and inappropriate antidiuretic hormone secretion (SIADH), are associated with abnormalities in water homeostasis. It has been shown that certain phytocompounds are beneficial to human health. Here, the effects of the Olive Leaf Extract (OLE) have been evaluated using in vitro and in vivo models. Confocal studies showed that OLE prevents the vasopressin induced AQP2 translocation to the plasma membrane in MCD4 cells and rat kidneys. Incubation with OLE decreases the AVP-dependent increase of the osmotic water permeability coefficient (Pf). To elucidate the possible effectors of OLE, intracellular calcium was evaluated. OLE increases the intracellular calcium through the activation of the Calcium Sensing Receptor (CaSR). NPS2143, a selective CaSR inhibitor, abolished the inhibitory effect of OLE on AVP-dependent water permeability. In vivo experiments revealed that treatment with OLE increases the expression of the CaSR mRNA and decreases AQP2 mRNA paralleled by an increase of the AQP2-targeting miRNA-137. Together, these findings suggest that OLE antagonizes vasopressin action through stimulation of the CaSR indicating that this extract may be beneficial to attenuate disorders characterized by abnormal CaSR signaling and affecting renal water reabsorption.

## Introduction

Olive tree leaves have been widely used as traditional remedies to cure several diseases, especially in Mediterranean countries^1^. Leaves are commonly consumed in the human diet as an extract or powder to prepare infusions or herbal tea. Chemical characterization analysis revealed that olive tree leaves contain several bioactive compounds that may exert beneficial effects in certain morbidities such as metabolic syndrome, dyslipidemia, and hypertension^2,3^. In patients with essential hypertension, OLE decreased blood pressure and slightly reduced glycemia and calcemia^4^. Numerous polyphenols such as oleuropein, hydroxytyrosol, and tyrosol have been found enriched in a green olive leaf extract (OLE)^5,6^ and they are known to be involved in the prevention of certain diseases characterized by oxidative stress. Therefore, in the last few years, the interest in investigating the potential beneficial effects of olive leaf extract is increasing among scientists in different fields of research. OLE displayed a significant antiproliferative effect in malignant mesothelioma cells by altering intracellular calcium dynamics, possibly targeting T-type Ca2+ channels^7^. Interestingly, OLE was found to exert antihypertensive effects on genetic hypertension in spontaneously hypertensive rats (SHR), related to the improvement of vascular function^8^. Excessive water reabsorption plays an important role in the pathogenesis of increased blood pressure^9^. In hypertensive patients, treated with olive leaf extract, a significant reduction of several inflammatory factors was found associated with a decrease in blood pressure^2^. Besides, excessive water retention characterizes several disorders such as liver cirrhosis, heart failure, and inappropriate antidiuretic hormone secretion (SIADH). Body water homeostasis is tightly controlled by the antidiuretic hormone vasopressin (AVP) that is released under the dehydrated condition^10^. AVP binds its cognate V2R receptor localized at the basolateral membrane of renal principal cells thereby activating the cAMP signal pathway that results in the translocation of the aquaporin-2 (AQP2) bearing vesicles from an intracellular pool to the apical plasma membrane where water reabsorption occurs. At a molecular level, several factors may modulate renal water balance. An elevated level of luminal calcium downregulated the short-term vasopressin-dependent AQP2 trafficking through the activation of the calcium-sensing receptor (CaSR)^11,12^. On the other hand, CaSR activation in conditionally immortalized renal proximal tubular epithelial cells, isolated from the human urine, induced a significant increase in cytosolic calcium and significantly reduced the increase in cAMP elicited by forskolin (FK), a direct activator of adenylate cyclase^13^. Moreover, in renal collecting duct MCD4 cells, stably expressing the water channel AQP2, stimulation of the CaSR attenuated the cAMP-dependent increase in AQP2 phosphorylation at serine 256 that is a pivotal event in the signal transduction cascade activated by vasopressin and that eventually increased water permeability^14,15^. Furthermore, in kidney slices from the inner medulla mouse kidney, activation of the CaSR with the calcimimetic NPS-R568 caused a significant increase in the AQP2-targeting miRNA-137, confirming a tight interplay between the CaSR signal pathway and AQP2-dependent water permeability^16,17^. In the present study, the effects of the olive leaf extract, obtained from the local *Coratina* cultivar, were evaluated on vasopressin-induced AQP2 function in renal collecting duct MCD4 cells stably expressing AQP2 and in dDAVP treated rats injected with the extract. We provide evidence that OLE treatment counteracts vasopressin response in renal cells, which might in part explain its diuretic effect. Interestingly, this effect appears to be related to OLE-induced activation of the CaSR, a receptor known to have a negative interplay with the vasopressin receptor^12^.

## Results

### Effects of OLE on vasopressin induced AQP2 function in MCD4 cells

**Figure 1:**
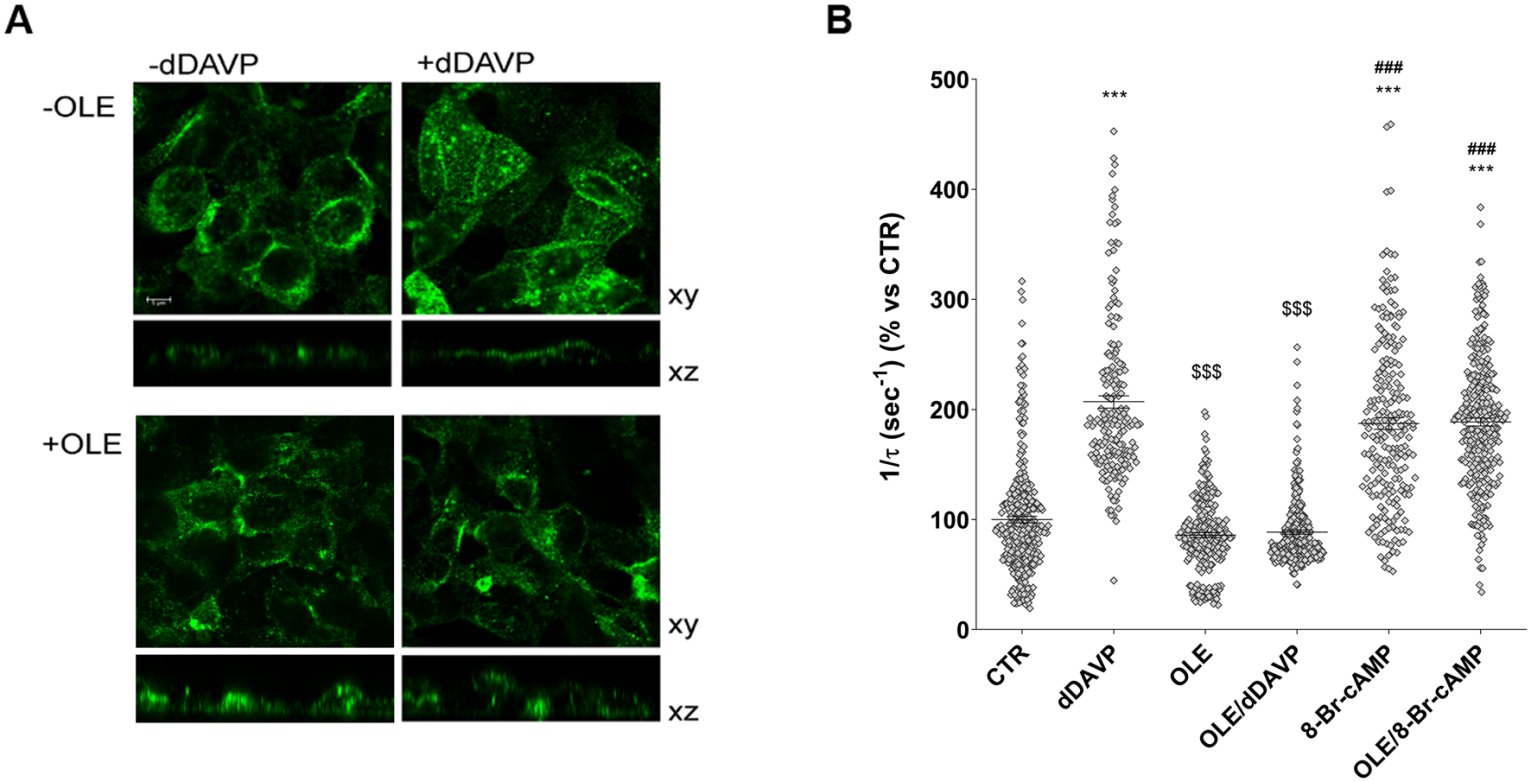
Effects of OLE on AQP2 localization and function in MCD4 cells. (**A**) Effect of OLE on AQP2 distribution in MCD4 cells. Cells were treated as described in the methods (left under basal conditions or treated with dDAVP (100 nM for 30 min), OLE (0.1 mg/ml O/N), or co-treated with OLE and dDAVP) and stained for AQP2 and subjected to confocal laser scanning microscope. Scale bar: 5 µm. (**B**) Osmotic water permeability in MCD4 cells was measured as reported in the methods. Cells were left under basal conditions or treated with dDAVP (100 nM for 30 min), OLE (0.1 mg/ml O/N), or co-treated with OLE and dDAVP, 8-Br-cAMP (500 µM for 45 min), or co-treated with OLE and 8-Br-cAMP. The temporal osmotic response is indicated as 1/τ (expressed in sec^−1^). Data are shown as single values in a scatter plot reporting means ± S.E.M (***p < 0.001 vs CTR; $$$p < 0.001 vs dDAVP; ###p < 0.001 vs OLE).

**Figure 2:**
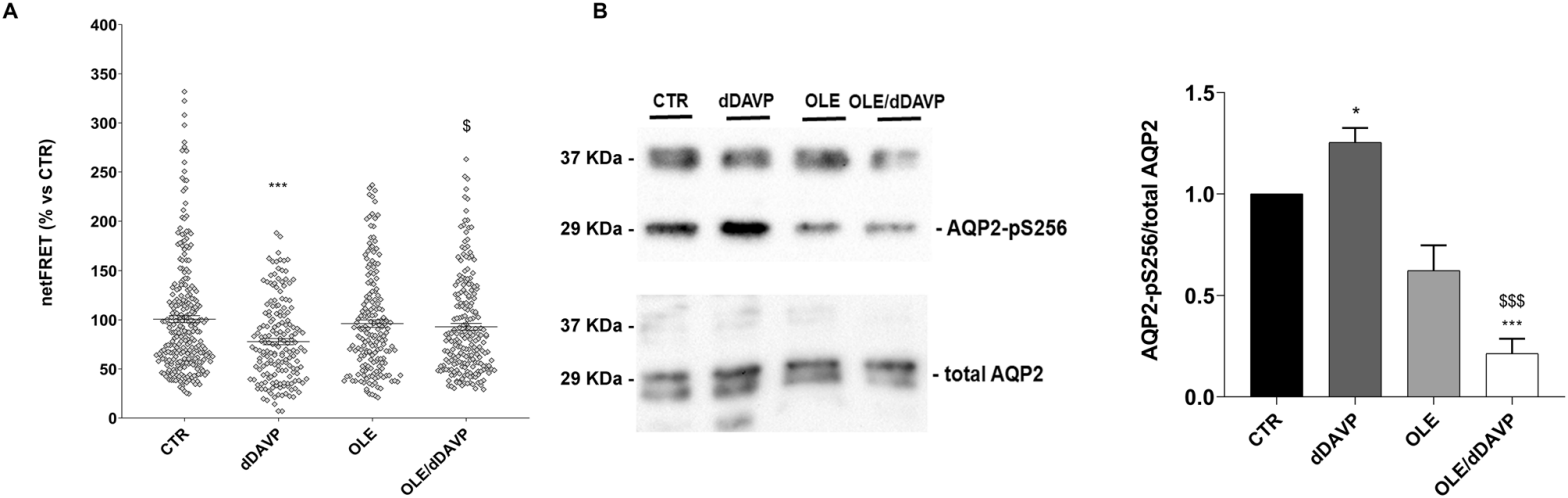
Effects of OLE on AQP2 phosphorylation and cAMP production in MCD4 cells. (**A**) Evaluation of cAMP production by FRET experiments in MCD4 cells transiently transfected with H96 as described in the “Materials and methods” section. Histogram reports netFRET measured in cells under basal conditions, treated with dDAVP (100 nM for 30 min), OLE (0.1 mg/ml O/N), or co-treated with OLE and dDAVP. The treatment with dDAVP displayed a significantly higher cAMP production depicted as a reduced netFRET signal. Data are shown as single values in a scatter plot reporting means ± S.E.M (***p < 0.001 vs CTR; $p < 0.05 vs dDAVP). (**B**) Lysates from MCD4 cells were subjected to immunoblotting using Abs against AQP2-pS256 and totalAQP2. Densitometric analysis of AQP2-pS256 bands normalized to total AQP2 bands (total AQP2) is reported in the histogram. Data are expressed as means ± S.E.M (*p < 0.05 or ***p < 0.001 vs CTR; $$$p < 0.001 vs dDAVP).

**Figure 3:**
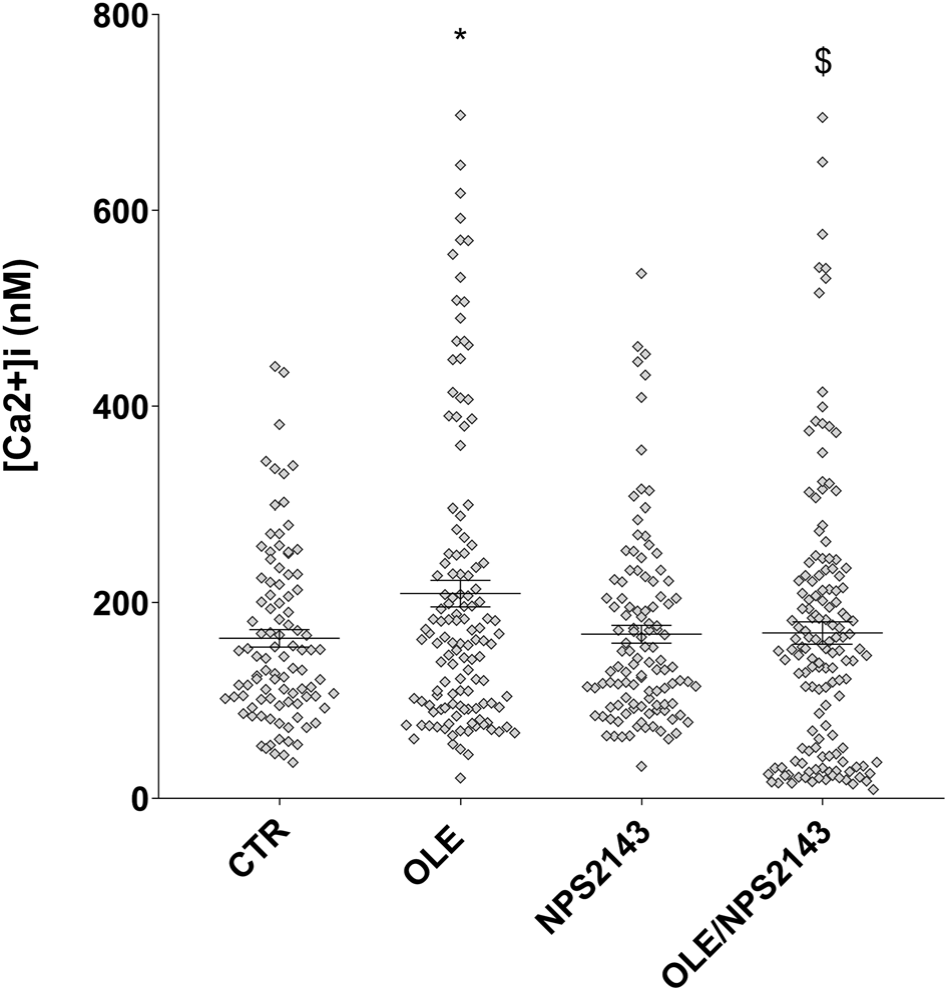
Effects of OLE on intracellular calcium concentration in MCD4 cells. MCD4 cells, loaded with Fura-2AM (4 μM for 15 min as described in methods), were stimulated with OLE (0.1 mg/ml O/N) in absence or in presence of NPS2143 (1 µM O/N). Intracellular calcium level was measured at steady-state and calibrated as described by Grynkiewicz ^57^. Each sample was calibrated by the addition of 5 µM ionomycin in presence of 1 mM EGTA (Rmin) followed by 5 µM ionomycin in 5 mM CaCl_2_ (Rmax). Data are shown as single values in a scatter plot reporting means ± S.E.M (*p < 0.05 vs CTR; $p < 0.05 vs OLE).

**Figure 4:**
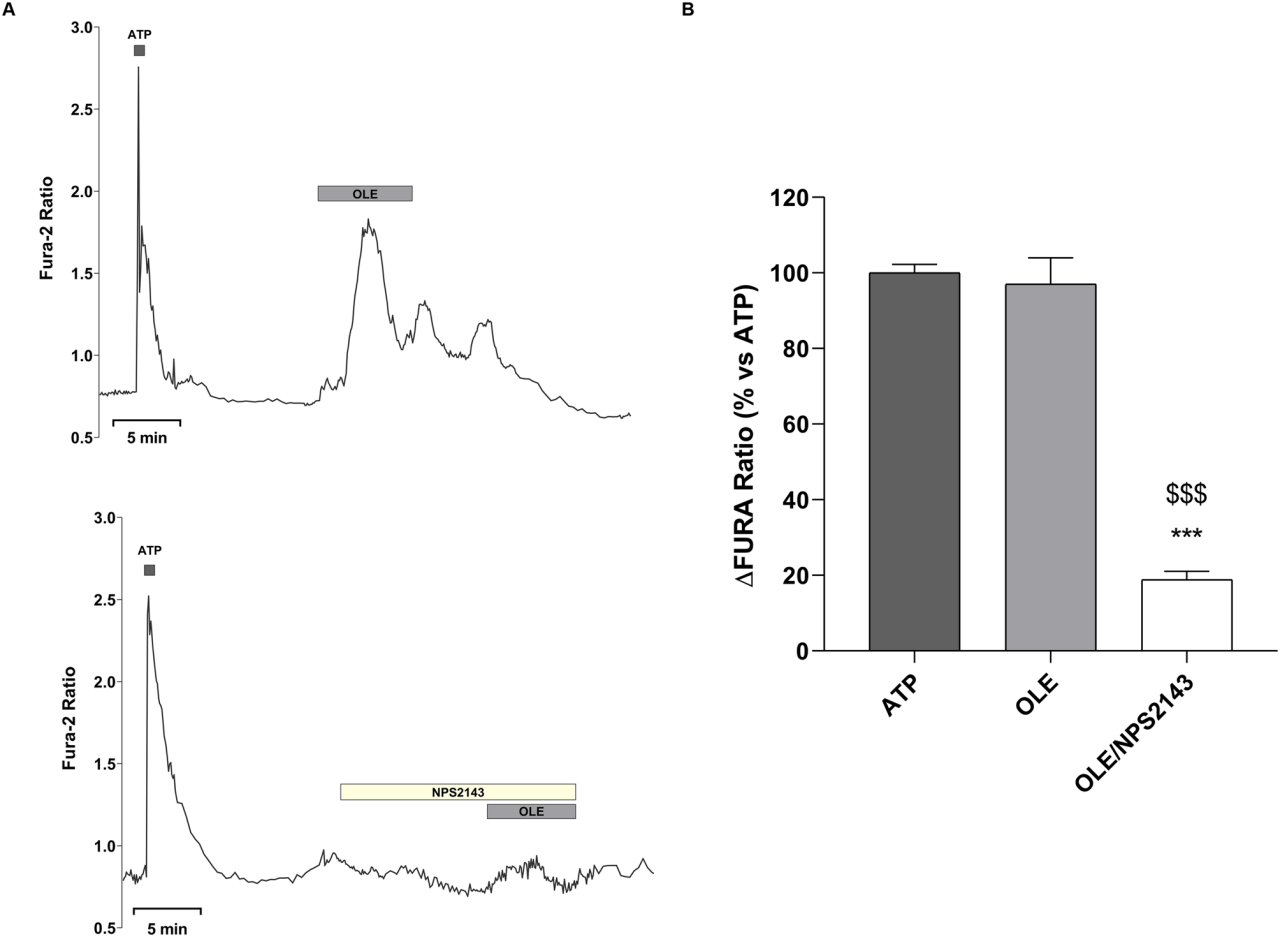
Effects of OLE on intracellular calcium oscillations in MCD4 cells. (**A**) MCD4 cells, loaded with Fura-2AM (4 μM for 15 min) as described in methods, were stimulated with OLE (1 mg/ml) in the absence or in the presence of NPS2143 (10 µM). Fluorescence ratio 340/380 nm was recorded and responses to OLE or OLE/NPS2143 were compared to that obtained after stimulation with a maximal dose of the calcium-mediated agonist ATP (100 μM) that was used as an internal control (100%). Each trace is representative of 3 different experiments with similar results. (**B**) Histogram shows the ΔFura Ratio percentage. Data are expressed as means ± S.E.M experiments (***p < 0.001 vs ATP; $$$p < 0.001 vs OLE).

**Figure 5:**
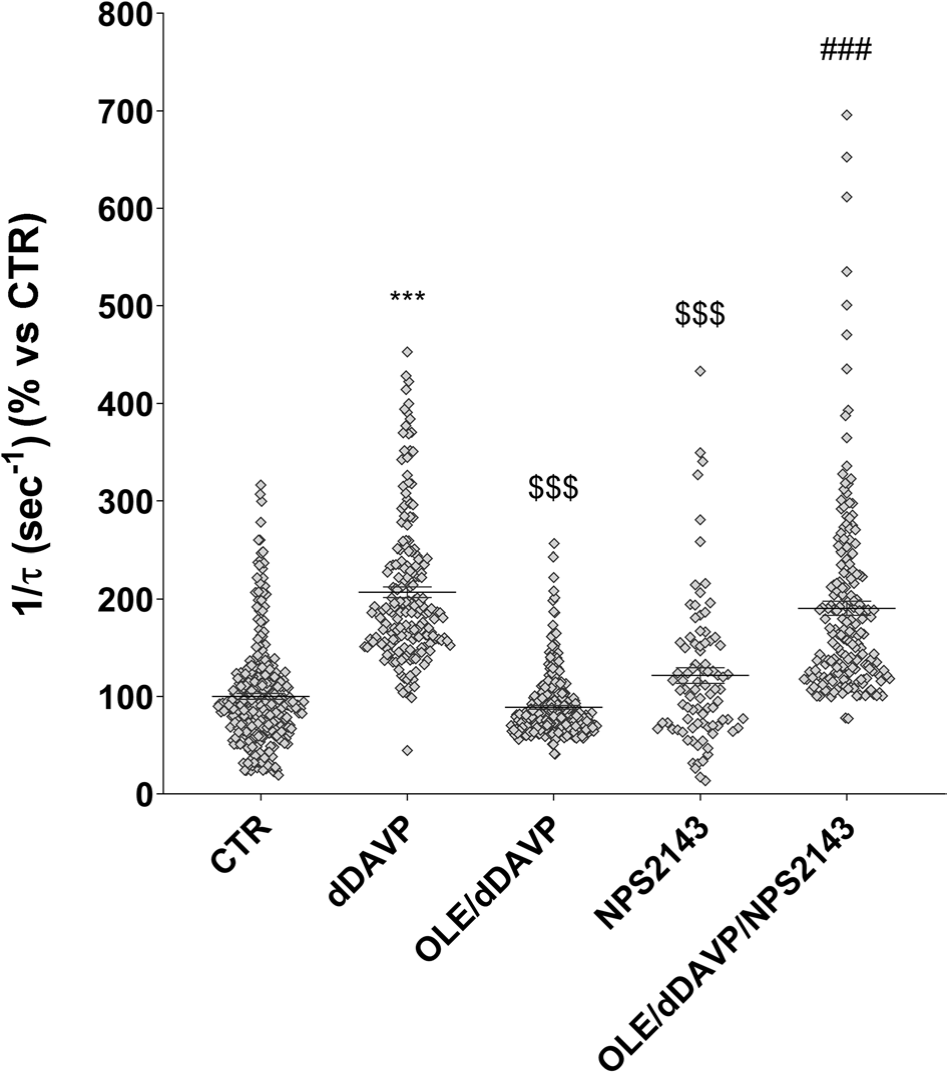
Effects of OLE on AQP2 function in MCD4 cells under CaSR inhibition by NPS2143. Osmotic water permeability was measured in MCD4 cells, loaded with Calcein-AM (10 μM for 45 min) as described in methods. Cells were stimulated with dDAVP (100 nM for 30 min), OLE (0.1 mg/ml O/N) and NPS2143 (1 µM O/N). Alternatively, cells were co-treated with OLE/dDAVP or OLE/dDAVP/NPS2143. The temporal osmotic response is indicated as 1/τ (expressed in sec^−1^). Data are shown as single values in a scatter plot reporting means ± S.E.M (***p < 0.001 vs CTR; $$$p < 0.001 vs dDAVP; ###p < 0.001 vs OLE/dDAVP).

To investigate the possible involvement of OLE on vasopressin-dependent AQP2 function, renal collecting duct MCD4 cells, stably expressing the vasopressin receptor 2 (V2R) and human AQP2, were used as an experimental model^18^. Confocal studies (Fig. 1A) revealed that, compared with dDAVP treated cells, in which AQP2 staining localized to the apical plasma membrane, treatment with OLE prevented the membrane localization of AQP2 induced by stimulation with dDAVP. Consistent with these observations, OLE treatment impaired the dDAVP-induced increase of the temporal osmotic response (indicated as 1/τ in Fig. 1B) (OLE/dDAVP = 88.70 ± 1.86%, n = 292 cells vs dDAVP = 206.8 ± 5.49%, n = 186 cells; p < 0.001). Altogether these findings suggested that treatment with OLE reduced principal cell permeability by preventing AQP2 translocation from an intracellular vesicle pool to the apical plasma membrane. Vasopressin-induced AQP2 trafficking is controlled by intracellular cAMP, which stimulated the cAMP-dependent kinase (PKA)^19^. Further, to evaluate the functionality of the V2R in the presence of OLE in terms of cAMP production, permeable 8-Br-cAMP was used as an external source of cAMP. Treatment with 8-Br-cAMP abolished the inhibitory effect elicited by OLE on osmotic water permeability (OLE/8-Br-cAMP = 188.7 ± 3.56%, n = 293 cells vs OLE = 85.93 ± 2.27%, n = 238 cells; p < 0.001). Therefore, to evaluate whether treatment with OLE regulates AQP2 trafficking by fine-tuning intracellular cAMP level, fluorescence resonance energy transfer (FRET) technology was applied. Compared to untreated cells (CTR), normalized netFRET signals are reduced with dDAVP stimulation (dDAVP = 77.86 ± 3.13%, n = 173 cells vs CTR = 100.00 ± 3.56%, n = 276 cells; p < 0.001; Fig. 2A), consistent with a significant increase of intracellular cAMP. Conversely, treatment with OLE prevented the dDAVP dependent decrease of netFRET signals consistent with a decrease of the vasopressin dependent cAMP release (OLE/dDAVP = 92.97 ± 3.46%, n = 211 cells vs dDAVP = 77.86 ± 3.13%, n = 173 cells; p < 0.05; Fig. 2A). Treatment with OLE alone did not affect the intracellular cAMP level compared with cells left under basal conditions (OLE = 96.15 ± 3.80%, n = 184 cells vs CTR = 100.00 ± 3.56%, n = 276 cells; p  < 0.05). Besides, Western Blotting studies (Fig. 2B) revealed that treatment with OLE significantly reduced the dDAVP induced increase of AQP2 phosphorylation at serine 256 (AQP2-pS256), indicating that AQP2 phosphorylation and trafficking, in response to OLE, are dependent on cAMP-PKA function. The phosphorylation level of AQP2-pS256 in response to OLE was not statistically different from the control even though it tended to be reduced. To gain insight into the molecular signals underlying the action of OLE, intracellular calcium dynamics were evaluated. MCD4 cells endogenously express a functional calcium-sensing-receptor (CaSR)^20^, which plays an important role in controlling AQP2 expression and trafficking^14^. Long term incubation with OLE (0.1 mg/ml) slightly increased the intracellular calcium level compared with untreated cells (Fig. 3; OLE = 209.0 ± 13.45 nM, n = 128 cells vs CTR = 163.3 ± 8.957 nM, n = 97 cells; p < 0.05). Co-incubation with OLE (0.1 mg/ml) and the selective NPS2143 (1 µM) antagonist of the CaSR abolished the OLE induced intracellular calcium mobilization (OLE/NPS2143 = 168.8 ± 11.43 nM, n = 144 cells vs OLE = 209.0 ± 13.45 nM, n = 128 cells; p < 0.05). To dissect further intracellular calcium signals in response to OLE, functional experiments were also performed under acute stimulation with OLE and NPS2143. Short term treatment with OLE (1 mg/ml) evoked specific calcium oscillations (Fig. 4A) that were prevented when cells were pretreated with the selective CaSR inhibitor NPS2143 (10 µM). Statistical analysis of the fluorescence responses (Fig. 4B) revealed that incubation with OLE increased cytosolic calcium by 97.06 ± 6.93% (vs ATP 100.00 ± 2.28%). Pretreatment with NPS2143 reduced the OLE induced intracellular calcium increase (OLE/NPS2143 = 18.87 ± 2.17%, n = 59 cells vs OLE = 97.06 ± 6.93%, n = 50 cells; p < 0.001). To deeper investigate whether the effect of OLE on AVP-dependent water reabsorption occurred through the activation of the CaSR signaling, functional studies were performed as previously shown (Fig. 1B). Interestingly, treatment with NPS2143 abolished the inhibitory effect of OLE on AVP-regulated osmotic water permeability (Fig. 5; OLE/dDAVP/NPS2143 = 190.4 ± 7.15%, n = 191 cells vs OLE/dDAVP = 88.70 ± 1.86%, n = 292 cells; p < 0.001), suggesting that treatment with OLE attenuated the AVP-dependent water reabsorption through the activation of the CaSR signaling. Compared to untreated cells, treatment with the only NPS2143 did not affect the osmotic water permeability (Fig. 5).

### Effects of OLE in the kidneys of rats injected with OLE

**Figure 6:**
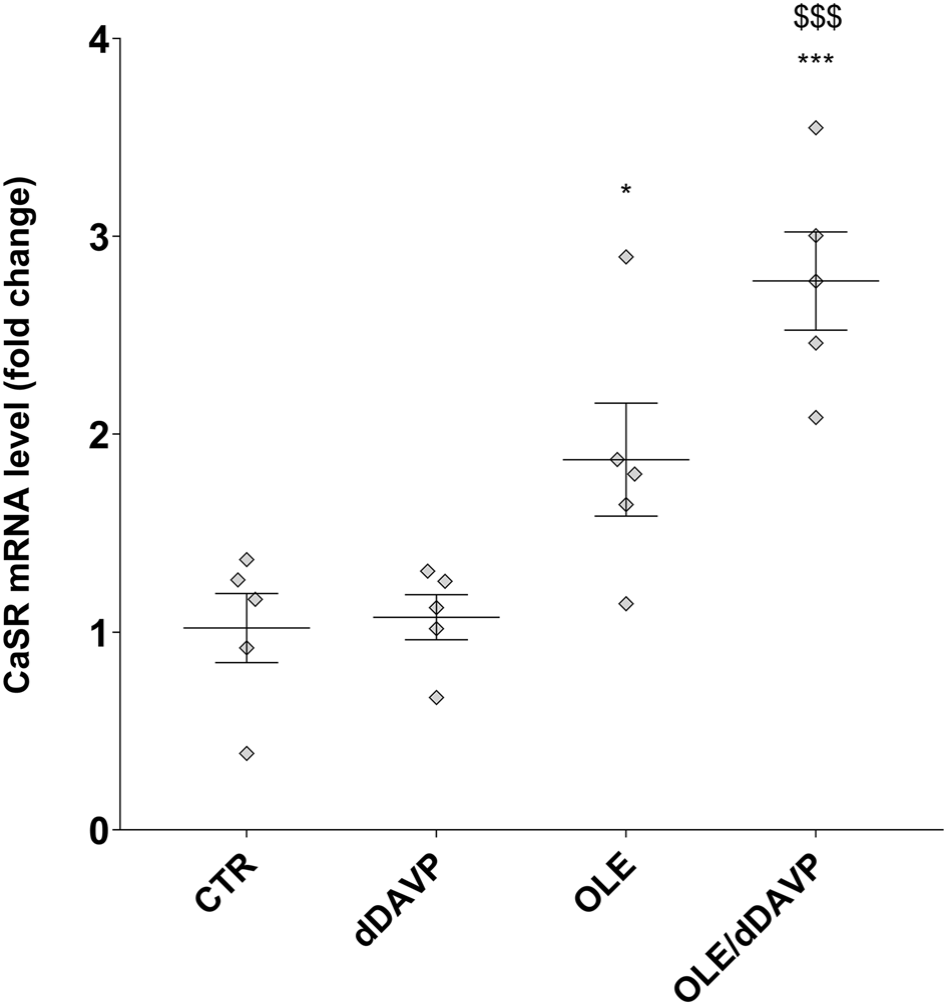
Effects of OLE on CaSR mRNA expression in rat kidneys. For the analysis of CaSR mRNA levels, RNA was extracted from control and treated rat kidneys as described in methods. Data are shown as single values in a scatter plot reporting means ± S.E.M (*p < 0.05 or ***p < 0.001 vs CTR; $$$p < 0.001 vs dDAVP).

**Figure 7:**
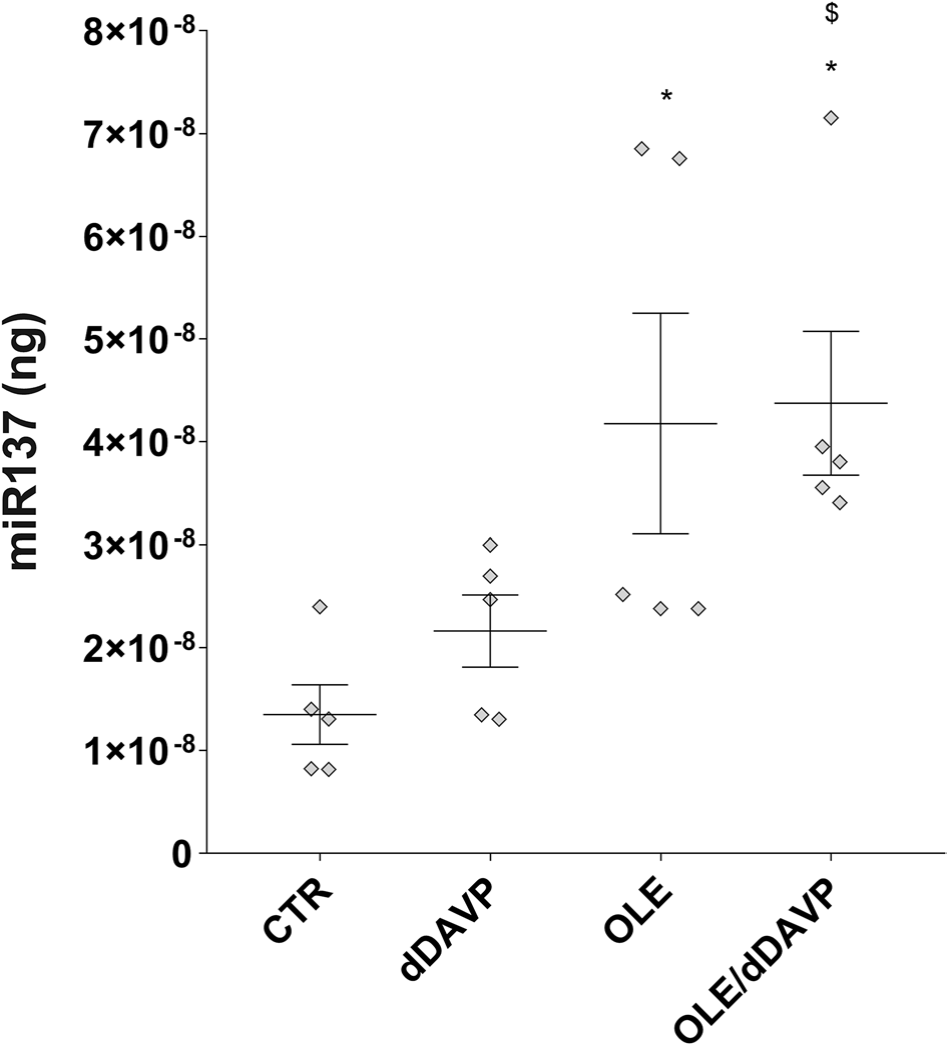
Effects of OLE on miR-137 expression in rat kidneys. miR-137 was evaluated in control and treated rat kidneys as described in methods. Total RNA was extracted from the inner medulla, and the miRNA cDNA Synthesis Kit was used to obtain cDNA synthesis, as described in methods. Synthetic RNA with 59-phospho miR-137, was synthesized and used to perform a calibration curve. Data from RT-PCR experiments were interpolated in the calibration line obtained with synthetic miRNA. Data are shown as single values in a scatter plot reporting means ± S.E.M (*p < 0.05 vs CTR; $p < 0.05 vs dDAVP).

**Figure 8:**
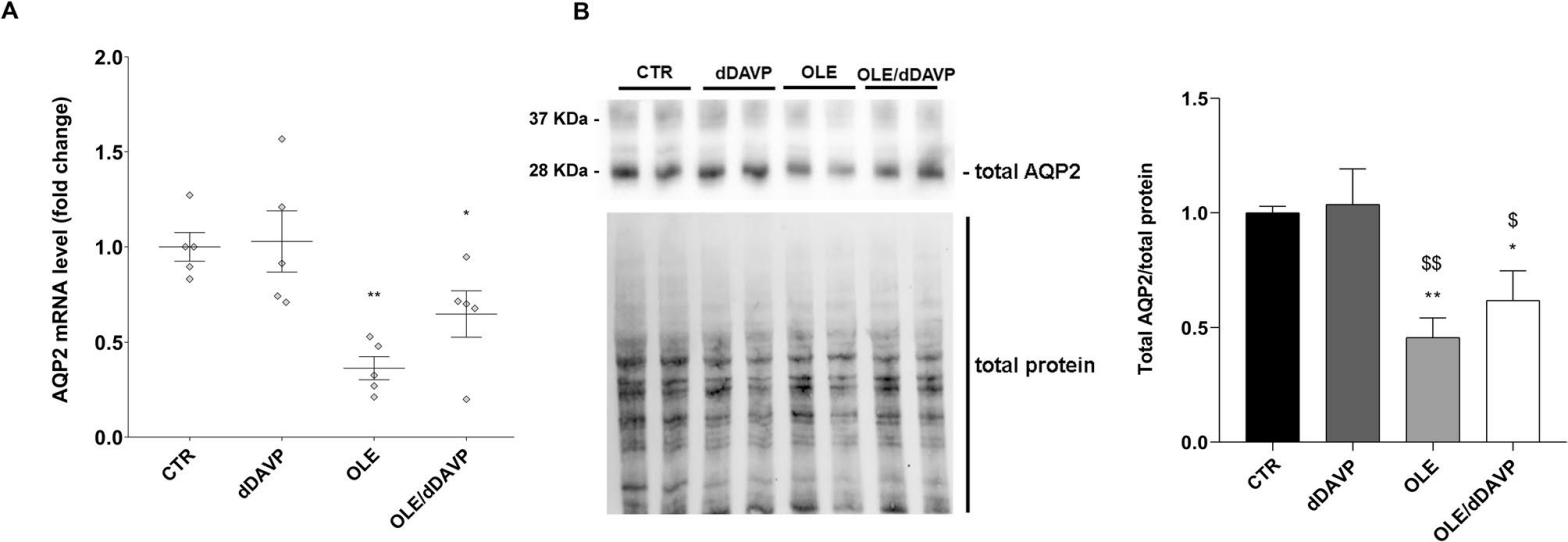
Effects of OLE on AQP2 mRNA and total protein expression in rat kidneys. (**A**) For the analysis of AQP2 mRNA levels, RNA was extracted from control and treated rat kidneys as described in methods. Data are shown as single values in a scatter plot reporting means ± S.E.M (*p < 0.05 or **p < 0.01 vs CTR). (**B**) Densitometric analysis of total AQP2 bands normalized to the total protein content is reported in the histogram. Data are expressed as means ± S.E.M (*p < 0.05 or **p < 0.01 vs CTR; $p < 0.05 or $$p < 0.01 vs dDAVP).

**Figure 9:**
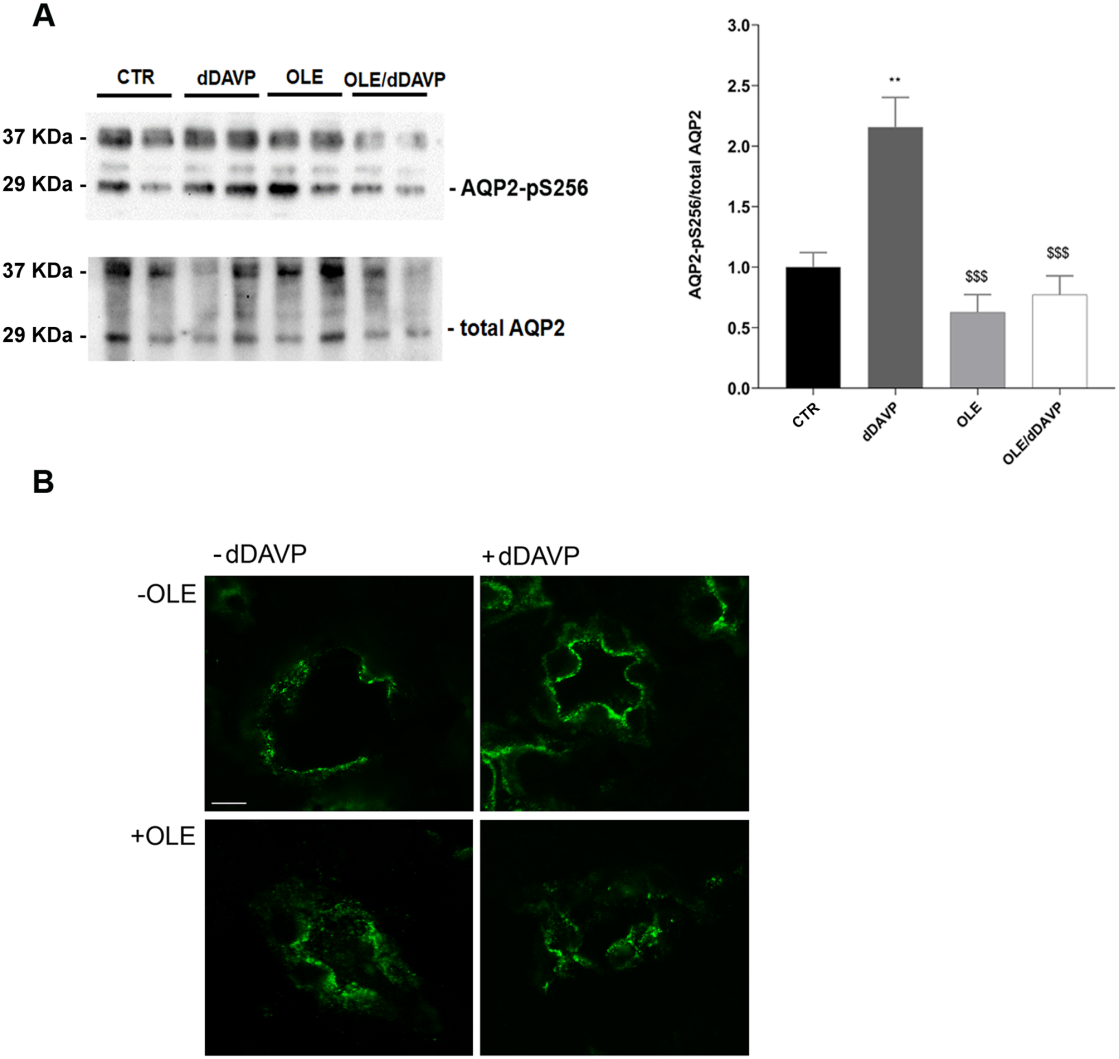
Effects of OLE on AQP2 phosphorylation and expression in rat kidneys. (**A**) Lysates from control and treated inner medulla kidney slices were subjected to immunoblotting using specific antibodies against AQP2-pS256 and total AQP2. Densitometric analysis of AQP2-pS256 bands normalized to total AQP2 bands (total AQP2) is reported in the histogram. Data are expressed as means ± S.E.M (**p < 0.01 vs CTR; $$$p < 0.001 vs dDAVP). (**B**) Rat kidney sections were stained for AQP2 and subjected to confocal laser scanning microscopy, as described in methods. Scale bar: 10 µm.

To further investigate the actions of OLE, in vivo studies have been performed. Specifically, rats have been injected with OLE (250 mg/kg/day) once a day for three days. Alternatively, rats were co-treated with OLE, for 3 days, and dDAVP, for 30 min. Evaluation of CaSR mRNA that was isolated from the renal collecting duct of rats and processed for real-time PCR (Fig. 6) revealed a significant increase in CaSR mRNA in OLE (OLE = 1.87 ± 0.29, n = 5 rats vs CTR = 1.02 ± 0.17, n = 5 rats; p < 0.05) and OLE/dDAVP (OLE/dDAVP = 2.77 ± 0.25, n = 5 rats vs CTR = 1.02 ± 0.17, n = 5 rats and dDAVP = 1.08 ± 0.11, n = 5 rats; p < 0.001) injected rats compared with control and dDAVP animals. CaSR signaling can regulate the expression level of selective miRNAs, which downregulated the expression level of AQP2^16^. Based on these findings, and considering that treatment with OLE attenuated AQP2 function, the level of miR-137, a known AQP2-targeting miR in the renal collecting duct, was evaluated. Compared to control rats (Fig. 7), the level of miR-137 in the renal collecting ducts of OLE (OLE = 4.18·10^–8^ ± 1.07·10^-8^ ng, n = 5 rats vs CTR = 1.35·10^–8^ ± 2.89·10^-9^ ng, n = 5 rats; p < 0.05) and OLE/dDAVP rats was increased indicating that treatment with OLE upregulates the CaSR signaling and the expression level of the miR-137 (OLE/dDAVP = 4.38·10^–8^ ± 47.01·10^–9^ ng, n = 5 rats vs CTR = 1.35·10^–8^ ± 2.89·10^-9^ ng, n = 5 rats; p < 0.01 and vs dDAVP = 2.16·10^–8^ ± 3.52·10^–9^ ng, n = 5 rats; p < 0.05). To verify whether the increase of miR-137 affected the expression level of AQP2, renal samples were processed for real-time PCR. Data (Fig. 8A) revealed that the expression levels of AQP2 mRNA were lower in OLE (OLE = 0.36 ± 0.06, n = 5 rats vs CTR = 1.00 ± 0.08, n = 5 rats; p < 0.01) and OLE/dDAVP (OLE/dDAVP = 0.65 ± 0.12, n = 5 rats vs CTR = 1.00 ± 0.08, n = 5 rats and dDAVP = 1.03 ± 0.16, n = 5 rats; p < 0.05) renal samples compared with specimens obtained from control animals. Besides, Western Blotting analysis (Fig. 8B) showed that treatment with OLE reduced the abundance of AQP2 regardless of dDAVP stimulation (OLE/dDAVP = 0.62 ± 0.13, n = 5 rats vs dDAVP = 1.04 ± 0.16, n = 5 rats; p < 0.05) indicating that the OLE-induced reduction of AQP2 mRNA expression level coincided with a decrease of AQP2 protein content. To evaluate the effect of OLE on AQP2 phosphorylation and trafficking in the kidney, western blotting analysis, and confocal studies were performed (Fig. 9). In OLE/dDAVP injected rats, phosphorylation of AQP2 at serine 256 was significantly reduced compared with renal tissues of dDAVP treated animals (OLE/dDAVP = 0.77 ± 0.16, n = 5 rats vs dDAVP = 2.16 ± 0.25, n = 5 rats; p < 0.001, Fig. 9A), thereby confirming the in vitro findings (Fig. 2B). Besides, confocal studies (Fig. 9B) of kidney sections revealed that exposure to OLE prevented the AQP2 membrane localization that instead was observed in dDAVP treated rats.

## Discussion

Calcium is an important intracellular messenger that modulates a plethora of cellular functions^21^. Abnormal calcium signaling may lead to several diseases including heart failure, cancer, and hypertension^22,23^. Specific control of the expression and the activity of proteins controlling multiple intracellular calcium signals has been proven successful to face numerous disorders and is therefore attractive for drug development. This study provides novel insights into the mechanism of action of OLE by showing its ability to modulate intracellular calcium signaling through the stimulation of the CaSR that is involved in fine-tuning the vasopressin induced AQP2 trafficking and expression^12,24^. Under physiological conditions, AQP2 plays an important role in controlling body water homeostasis^25^. Stimulation of the intracellular machinery, leading to localization of AQP2 at the apical plasma membrane, results in abnormal water retention and consequent hyponatremia as in the syndrome of inappropriate antidiuretic hormone (SIADH) secretion, liver cirrhosis, and congestive heart failure^26–28^. In a mouse model of SIADH, associated with polycystic kidney disease 1 haploinsufficiency, the basal intracellular calcium level was significantly reduced compared to the level measured in the collecting ducts of wild type mice^29^. Low intracellular calcium downregulated the activity of certain enzymes including the protein phosphatase PP2A resulting in the upregulation of AQP2 phosphorylation at serine 256^30,31^. By contrast, activation of the CaSR with the calcimimetic NPS-R568 increased the intracellular calcium concentration and reduced the cAMP level in PKD1 deficient cells^32^. Importantly, cytosolic calcium can downregulate the calcium-inhibitable adenylated cyclase^33^ thereby fine-tuning the intracellular level of cAMP. In MCD4 cells, indeed, stimulation of the CaSR with NPS-R568 decreased AQP2-pS256 through the inhibition of the adenylate cyclase^14^. In this study, treatment with OLE prevented the vasopressin dependent increase of the cAMP and AQP2-pS256, possibly secondary to an increase in the intracellular calcium concentration. Of note, exposure to an external source of cAMP, using permeable 8-Br-cAMP, significantly counteracted the inhibitory effect of OLE on the V2R pathway.

An elevated level of cytosolic calcium may lead to cell death and apoptosis. However, a sustained increase in intracellular calcium from 250 nM to greater than 600 nM promoted neuronal survival^34^. In MCD4 cells, treatment with OLE at 0.1 mg/ml does not display a cytotoxic effect^35^. At this concentration, OLE (0.1 mg/ml) slightly increased the intracellular calcium level. Calcium imaging revealed that acute stimulation with OLE caused a transient increase of intracellular calcium through the activation of the CaSR that is abolished when cells were preincubated with the selective CaSR antagonist NPS2143. In renal proximal tubule cells, allosteric activation of the CaSR with I-ornithine reduced the ROS production thereby downregulating the mitochondrial oxidative stress that caused cell apoptosis^36^. Moreover, our previous study showed that the expression of the gain-of-function variants of the CaSR reduced ROS release and S-glutathionylation of AQP2^37^. Incubation with OLE decreased the ROS generation in MCD4 cells^35^ showing its antioxidant ability. Nevertheless, at moment it’s unclear whether OLE affects AQP2-S-glutathionylation. Numerous studies revealed that OLE or oleuropein, which is the main constituent of OLE, may have protective roles by inhibiting cell death^38,39^. However, dose-dependent effects have been described in rats receiving OLE. Beneficial responses were obtained at doses of 250 and 500 mg/kg. By contrast, at higher concentrations, treatment with OLE may have harmful effects^38^ possibly due to the pro-oxidant actions of several phytocompounds. In this study, rats were injected with OLE at a dose of 250 mg/kg for three days showing upregulation of CaSR. The expression level of the CaSR is increased by certain compounds that act as allosteric activators of the receptor. Calcimimetics, indeed, decreased the vascular calcifications by increasing the biosynthesis of the CaSR in human vascular smooth muscle cells^40^. In this contest, it cannot be excluded the possibility that certain compounds in OLE may function as allosteric modulators of the CaSR. On another hand, stimulation with vasopressin may lead to IL-6 release^41^ that may contribute to increasing the expression level of the CaSR^42^.

In the kidney, stimulation of the CaSR signaling is associated with a downregulation of AQP2 expression possibly through the miR-137 generation. miRNAs are pivotal posttranscriptional modulators. Several vasopressin-dependent miRNAs targeting AQP2 expression have also been described^43^. Transfection with miR-32 and miR-137 significantly reduced the expression level of AQP2 in mpkCCDc14 cells^43^. Interestingly, OLE can attenuate inflammatory signals and exert protective effects by modulating the expression level of several miRNA^44^. In particular, in glioblastoma multiforme cells, OLE promoted the expression of different miRNA including miR-137^45^ that is involved in the downregulation of Akt/mTOR signaling pathway ^46^. Of note, treatment with oleuropein prevents Akt/mTOR signaling through the activation of calcium-induced CAMK and AMPK^47^. Activation of CaSR with NPS-R568 activates AMPK and reduces mTOR activity in human proximal tubular cells^32^. Consistent with these findings, incubation with OLE increases cytosolic calcium, downregulates PKA activity, and reduces the cyst size in a 3D-cell culture model^48^. Together these findings suggest that OLE may be useful in treating disorders characterized by dysregulation of intracellular calcium dynamics related to the downregulation of CaSR signaling.

Olive leaf extract consists of several bioactive components, including polyphenols, fibers, and minerals^49^. At the moment, it is not clear if one compound or a synergic combination of phytocompounds in OLE may be beneficial in modulating intracellular responses. The extract used in this study has been applied as a possible food additive^5^ and it is therefore important to define the physiological impact of the extract itself considering that several phenols, including hydroxytyrosol, may be filtered by the kidney and recovered in the urine^50^. Further studies will provide the efficacy of specific components that may actively regulate intracellular calcium signaling through the CaSR. To conclude, this study provides evidence that OLE may be considered a novel potential adjuvant useful to mitigate disorders characterized by a reduction of CaSR expression as well as signaling and affecting renal water permeability.

## Materials and methods

### Chemicals and antibodies

All chemicals and antibiotics were purchased from Merck (Merck KGaA, Darmstadt, Germany). Calcein-AM, Fura-2-AM, cell culture media, fetal bovine serum (FBS), and Super Signal West Pico Chemiluminescent Substrates were bought from Thermo Fisher Scientific (Thermo Fisher Scientific, Waltham, MA, U.S.A.). Aquaporin-2 (AQP2) was detected using specific antibodies (C-tail Ab) raised against a synthetic peptide corresponding to the last 15 C-terminal aminoacids of human AQP2^51^. AQP2-pS256 antibodies were kindly gifted by Prof Peter Deen. Secondary goat anti-rabbit horseradish peroxidase–coupled antibodies were obtained from Merck (Merck KGaA, Darmstadt, Germany). Secondary goat-anti-rabbit antibody coupled to Alexa-488 was purchased from Thermo Fisher Scientific (Thermo Fisher Scientific, Waltham, MA, U.S.A.).

### Phenolics-rich extract production and chemical characterization

The production of a phenolics-rich extract from olive leaves was carried out as reported in Ranieri et al.^35^. After milling with a blender (Waring-Commercial, Torrington, CT, USA), ddH2O water was added (ratio 1/20, w/v) and then subjected to ultrasound (CEIA, Viciomaggio, Italy) three times, for 30 min at 35 ± 5 °C each time. Finally, the extracts were filtered through filter paper, lyophilized, and stored at -20 °C. The obtained extracts were filtered with nylon filters of 0.45 µm (Merck KGaA, Darmstadt, Germany) and used for chemical characterization. The total phenol content, the antioxidant activity, and the single phenolic compounds identification were performed according to Difonzo et al.^5^. The OLE showed a content of polyphenols, determined by Folin-Ciocalteu, equal to 195 mg·g^-1^ gallic acid equivalents (GAE) and an antioxidant activity, determined by ABTS (2,2′-azino-bis(3-ethylbenzothiazoline-6-sulfonic acid diammonium salt), accounting for 750 µmol TE (Trolox equivalents) g^-1^.

### Cell culture and treatments

Mouse cortical collecting duct MCD4 cells, stably expressing human AQP2 and the chimeric V2R-Rluc were used as an experimental model^18^. Cells were grown in a 1:1 mixture of Dulbecco’s modified Eagle’s medium and F-12 supplemented with 5% (v/v) fetal bovine serum, 1% (v/v) L-glutamine and 1% penicillin/streptomycin, 5 µM dexamethasone, 400 µg/ml G418 (for AQP2 resistance) and 1 µg/ml puromycin (for V2R-Rluc resistance), in a humidified atmosphere of 5% CO_2_ at 37 °C. MCD4 cells were left under basal condition (CTR) or long-term treated with OLE at 0.1 mg/ml O/N, dDAVP at 100 nM for 30 min or co-treated with OLE and dDAVP or NPS2143 at 1 µM O/N. Alternatively, cells were exposed to 8-Br-cAMP at 500 µM for 45 min. Short term experiments were carried out with OLE at 1 mg/ml for 5 min and NPS2143 at 10 µM for 15 min. Experiments were performed 3–5 independent times using cells from different passages.

### Animal model and treatments

All procedures were accomplished in agreement with the Danish National Guidelines for the care and handling of experimental animals and the published guidelines of the National Institutes of Health. The protocols were approved by the Institute of Clinical Medicine, Aarhus University, according to the licenses for the use of experimental animals issued by the Danish Ministry of Justice (Approval number 2015-15-0201-00658). Studies were performed on adult male Wistar rats with a starting weight of 200–225 g. Animals were maintained on a standard rodent diet (Altromin, Lage, Germany) and had free access to tap water. During the experiments, rats were housed in groups of two per cage, with a 12:12 h light–dark cycle, a temperature of 21 ± 2 °C, and a humidity of 55 ± 2%.

Five rats were treated with subcutaneous injection of 1 ng dDAVP (Sigma-Aldrich, Glostrup, Denmark) in 200 µl saline/animal, and five vehicle-treated rats served as controls. After 30 min, the rats were killed, and the kidneys were processed as described below.

### Cells and IMCD lysates

Cells were grown on 60-mm dishes and resuspended in a buffer containing 220 mM mannitol, 70 mM sucrose, 0.5 M EGTA pH 8.0, 0.5 M EDTA pH 8.0, 1 M Tris–HCl pH 7.4 in the presence of proteases (1 mM PMSF, 2 mg/ml leupeptin, and 2 mg/ml pepstatin A) and phosphatases (10 mM NaF and 1 mM sodium orthovanadate) inhibitors. Alternatively, kidney sections of approximately 0.5 mm were prepared and equilibrated for 10 min in a buffer containing 118 mM NaCl, 16 mM Hepes, 17 mM Na-Hepes, 14 mM glucose, 3.2 mM KCl, 2.5 mM CaCl2, 1.8 mM MgSO4, and 1.8 mM KH2PO4 (pH 7.4). Renal papilla were minced with scissors in the same buffer in the presence of proteases (1 mM PMSF, 2 mg/ml leupeptin, and 2 mg/ml pepstatin A) and phosphatases (10 mM NaF and 1 mM sodium orthovanadate) inhibitors. After sonication (60 kHz for 5 s), lysates were centrifuged at 12,000 × g for 10 min. The supernatants were collected and used for immunoblotting studies^18^.

### Confocal microscopy

Confocal studies were performed as previously described^31^. Briefly, cells were grown on cell culture PET inserts and treated as described above. Alternatively, kidney sections obtained from control, OLE, dDAVP, and OLE with dDAVP treated rats were subjected to immunofluorescence experiments as previously described^31^. Images were obtained with a confocal laser-scanning fluorescence microscope Leica TCS SP2 (Leica Microsystems, Heerbrugg, Switzerland).

### Gel electrophoresis and immunoblotting

Proteins were separated on 12% stain-free polyacrylamide gels (Bio-Rad Laboratories, Inc., Hercules, CA, U.S.A.) under reducing conditions as previously described^35,52^. Briefly, protein bands were transferred onto Immobilon-P membranes (Merck KGaA, Darmstadt, Germany) and immunoblotted using anti-AQP2 (Pre-C-tail Ab) and anti-AQP2-pS256 antibodies. Immunoreactive signals were obtained using Super Signal West Pico Chemiluminescent Substrates with Chemidoc System (Bio-Rad Laboratories, Inc., Hercules, CA, U.S.A.). Bands were normalized to total protein using stain-free technology (Bio-Rad Laboratories, Inc., Hercules, CA, U.S.A.). Densitometry analysis was performed using ImageLab (Bio-Rad Laboratories, Inc., Hercules, CA, U.S.A.) and analyzed using GraphPad Prism (GraphPad Software, San Diego, CA, USA).

### Water permeability assay

Osmotic water permeability was measured as previously described^14,53^. Briefly, MCD4 cells were grown on 40-mm glass coverslips. After treatments, cells were loaded with 10 μM membrane-permeable Calcein-AM for 45 min at 37 °C, 5% CO_2_ in DMEM/F-12. Coverslips were mounted in a perfusion chamber (FCS2 Closed Chamber System, BIOPTECHS, Butler, U.S.A.). Measurements were performed using an inverted TE2000-S microscope (Nikon Eclipse microscope, Tokyo, Japan) equipped for single-cell fluorescence measurements and imaging analysis. The Calcein-AM loaded samples were excited at 490 nm. Fluorescence measurements, following isosmotic (140 mM NaCl, 5 mM KCl, 1 mM MgCl_2_, 1 mM CaCl_2_, 10 mM Hepes sulfonic acid, 5 mM Glucose, pH 7.4) or hyperosmotic (isosmotic solution added with 135 mM Mannitol) solutions, were carried out using Metafluor software (Molecular Devices, MDS Analytical Technologies, Toronto, Canada). Time course fluorescence data after perfusing cells with iso- and hyperosmotic solutions were recorded. The time course of cell shrinkage was measured as time constant (*Ki*, expressed as 1/τ, sec^-1^), a parameter correlated to the water permeability, obtained by fitting data with an exponential function analyzed using GraphPad Prism (GraphPad Software, San Diego, CA, USA).

### Fluorescence resonance energy transfer measurements

To evaluate intracellular cAMP changes, fluorescence resonance energy transfer (FRET) technology was applied. For FRET experiments, cells were transfected with a plasmid encoding the H96 sensor containing the cAMP-binding consensus motif of EPAC1 embedded between the cyan fluorescent protein (CFP) and cp^173^Venus‐Venus as previously shown^32,53,54^. The plasmid encoding the H96 probe was a gift from Dr. K. Jalink. Visualization of ECFP- and/or EYFP-expressing cells and detection of FRET was performed using an inverted TE2000-S microscope (Nikon Eclipse microscope, Tokyo, Japan). Each image was corrected and analyzed as previously shown^55^.

### Intracellular calcium measurements

Intracellular calcium measurements were performed as previously shown^56^. Briefly, MCD4 cells were loaded with 4 µM Fura-2AM for 15 min at 37 °C in DMEM/F-12. Ringer’s solution was used to perfuse cells during the experiment containing 140 mM NaCl, 5 mM KCl, 1 mM MgCl_2_, 10 mM HEPES, 5 mM Glucose, 1.8 mM CaCl_2_, pH 7.4. In fluorescence measurements, the coverslips with dye-loaded cells were mounted in a perfusion chamber (FCS2 Closed Chamber System, BIOPTECHS, Butler, U.S.A.). Measurements were performed using an inverted TE2000-S microscope (Nikon Eclipse microscope, Tokyo, Japan). The ratio of fluorescence intensities at 340 and 380 nm was plotted and calculated as the change in fluorescence. In particular, stimulation with OLE (1 mg/ml) or NPS2143 (10 µM) was compared in the same cell type to those obtained after stimulation with a maximal dose of the calcium-mediated agonist ATP (100 µM) that was used as an internal control (100%).

Intracellular calcium level was measured at steady-state and calibrated as described by Grynkiewicz^57^. OLE and NPS2143 was used at long term at 0.1 mg/ml and 1 µM, respectively and each sample was calibrated by the addition of 5 µM ionomycin in presence of 1 mM EGTA (R_min_) followed by 5 µM ionomycin in 5 mM CaCl_2_ (R_max_).

### Real-time PCR analysis of AQP2 and CaSR mRNA in control and treated rats

Real-time PCR experiments were performed to measure the relative expression of mRNA in inner medulla collecting duct (IMCD) isolated from control and treated rat kidney papillae. Kidney slices of about 0.5 mm were made and equilibrated for 10 min in a buffer containing 118 mM NaCl, 16 mM Hepes, 17 mM Na-Hepes, 14 mM glucose, 3.2 mM KCl, 2.5 mM CaCl2, 1.8 mM MgSO4, and 1.8 mM KH2PO4 (pH 7.4). The renal papilla were isolated under stereo-microscope. Specimens from control and treated rats were then minced with a scissor directly in Trizol (Thermo Fisher Scientific, Waltham, MA, U.S.A.). Reverse transcription was performed on 2.5 µg of total RNA using SuperScriptVilo Master Mix (Thermo Fisher Scientific, Waltham, MA, U.S.A.), in accordance with the manufacturer suggestions (25 ºC for 10 min; 42 ºC for 60 min; 85 ºC for 5 min). Real-time PCR amplification was performed by using TaqMan Fast Advanced Master Mix with CaSR, AQP2 and GAPDH assays (Thermo Fisher Scientific, Waltham, MA, U.S.A.) in StepOne Real-Time PCR System (Thermo Fisher Scientific, Waltham, MA, U.S.A.), setting the thermal cycling conditions as specified by manufacturer (95 ºC for 20 s; 40 cycles alternatively at 95 ºC for 1 s and 60 ºC for 20 s). Results were expressed as 2^-ΔΔCt^ values (relative quantification) with ΔΔCt = (Ct target—Ct GAPDH) treated—(Ct target—Ct GAPDH) Sham^16^.

### miRNA-137 evaluation in control and Treated rats

miRNA-137 content in control and treated rat inner medulla collecting duct was evaluated using TaqMan Advanced miRNA Assays (has-miR-137; Assay ID: 477904_mir; Thermo Fisher Scientific, Waltham, MA, U.S.A.), which enabled highly sensitive and specific quantification of mature miRNA using quantitative PCR. Kidney slices of about 0.5 mm were made and equilibrated for 10 min in a buffer containing 118 mM NaCl, 16 mM Hepes, 17 mM Na-Hepes, 14 mM glucose, 3.2 mM KCl, 2.5 mM CaCl2, 1.8 mM MgSO4, and 1.8 mM KH2PO4 (pH 7.4). The renal papilla were isolated under stereo-microscope. Specimens from control and treated rats were then minced with a scissor directly in Trizol (Thermo Fisher Scientific, Waltham, MA, U.S.A.) to extract total RNA. TaqMan Advanced miRNA cDNA Synthesis Kit (Catalog n°: A25576; Thermo Fisher Scientific, Waltham, MA, U.S.A.) was used to obtain cDNA synthesis, according to the protocol provided by the manufacturer (Poly(A) tailing reaction; ligation reaction; reverse transcription reaction; miR-Amp reaction). The synthetic RNA (UUAUUGCUUAAGAAUACGCGUAG) with 59-phospho was synthesized by Thermo Fisher Scientific and used to perform a calibration curve to interpolate miRNA sample values and to obtain a precise evaluation (in nanograms) of miR-137 content in samples^16^, by using GraphPad Prism (GraphPad Software, San Diego, CA, USA).

### Statistical analysis

All values are reported as means ± S.E.M. Statistical analysis was performed by one-way ANOVA followed by Newman–Keuls multiple comparisons test with *p < 0.05 considered statistically different^58^.

### Statements, ethical approval and consent to participate

Animal studies were performed in agreement with the Danish National Guidelines for the care and handling of experimental animals and the published guidelines of the National Institutes of Health. Animal care ethics committee of Institute of Clinical Medicine, Aarhus University approved the study protocols, according to the licenses for the use of experimental animals issued by the Danish Ministry of Justice (Approval number 2015-15-0201-00658). The authors confirm that all methods were carried out in accordance with relevant guidelines and regulations. Moreover, the authors confirm that the study was carried out in compliance with the ARRIVE guidelines.

## Supplementary Information

Supplementary Information

## Abbreviations

OLE: Olive leaf extract
AVP: Vasopressin
AQP2: Aquaporin-2
SIADH: Inappropriate antidiuretic hormone secretion
CaSR: Calcium sensing receptor
SHR: Spontaneously hypertensive rats
cAMP: Cyclic adenosine monophosphate
FK: Forskolin
FBS: Fetal bovine serum
V2R: Vasopressin receptor 2
dDAVP: Desmopressin
