# Supplementary material for: Olive Leaf Extract (OLE) impaired vasopressin-induced aquaporin-2 trafficking through the activation of the calcium-sensing receptor
Source: Sci Rep. 2021 Feb 25;11:4537. doi: 10.1038/s41598-021-83850-5 (PMC7907100; doi:10.1038/s41598-021-83850-5)
Supplement: Supplementary file 1 — Supplementary Information [file 41598_2021_83850_MOESM1_ESM.docx]

**Olive Leaf Extract (OLE) impaired vasopressin-induced aquaporin-2 trafficking through the activation of the Calcium-Sensing Receptor**

Marianna Ranieri^1*^, Annarita Di Mise^1^, Mariangela Centrone^1^, Mariagrazia D’Agostino^1^, Stine Julie Tingskov^2^, Maria Venneri^1^, Tommaso Pellegrino^1^, GrazianaDifonzo^3^, Francesco Caponio^3^, Rikke Norregaard^2^, Giovanna Valenti^1^, Grazia Tamma^1*^

*^1^Deparent of Biosciences, Biotechnologies, and Biopharmaceutics, University of Bari Aldo Moro, Bari, Italy*

*^2^Department of Clinical Medicine, Aarhus University, Denmark*

*^3^Department of Soil, Plant and Food Sciences, University of Bari Aldo Moro, Bari, Italy*

*To whom correspondence should be addressed:

Corresponding author: Grazia Tamma, Ph.D., Department of Bioscience, Biotechnology and Biopharmaceutics, University of Bari Aldo Moro, Via Orabona 4, 70125 Bari-Italy Tel: +39 080 5442388 Email: [grazia.tamma@uniba.it](mailto:grazia.tamma@uniba.it);

Co-corresponding author: Marianna Ranieri, Ph.D., Department of Bioscience, Biotechnology and Biopharmaceutics, University of Bari Aldo Moro, Via Orabona 4, 70125 Bari-Italy Tel: +39 080 5443334 Email: marianna.ranieri@uniba.it


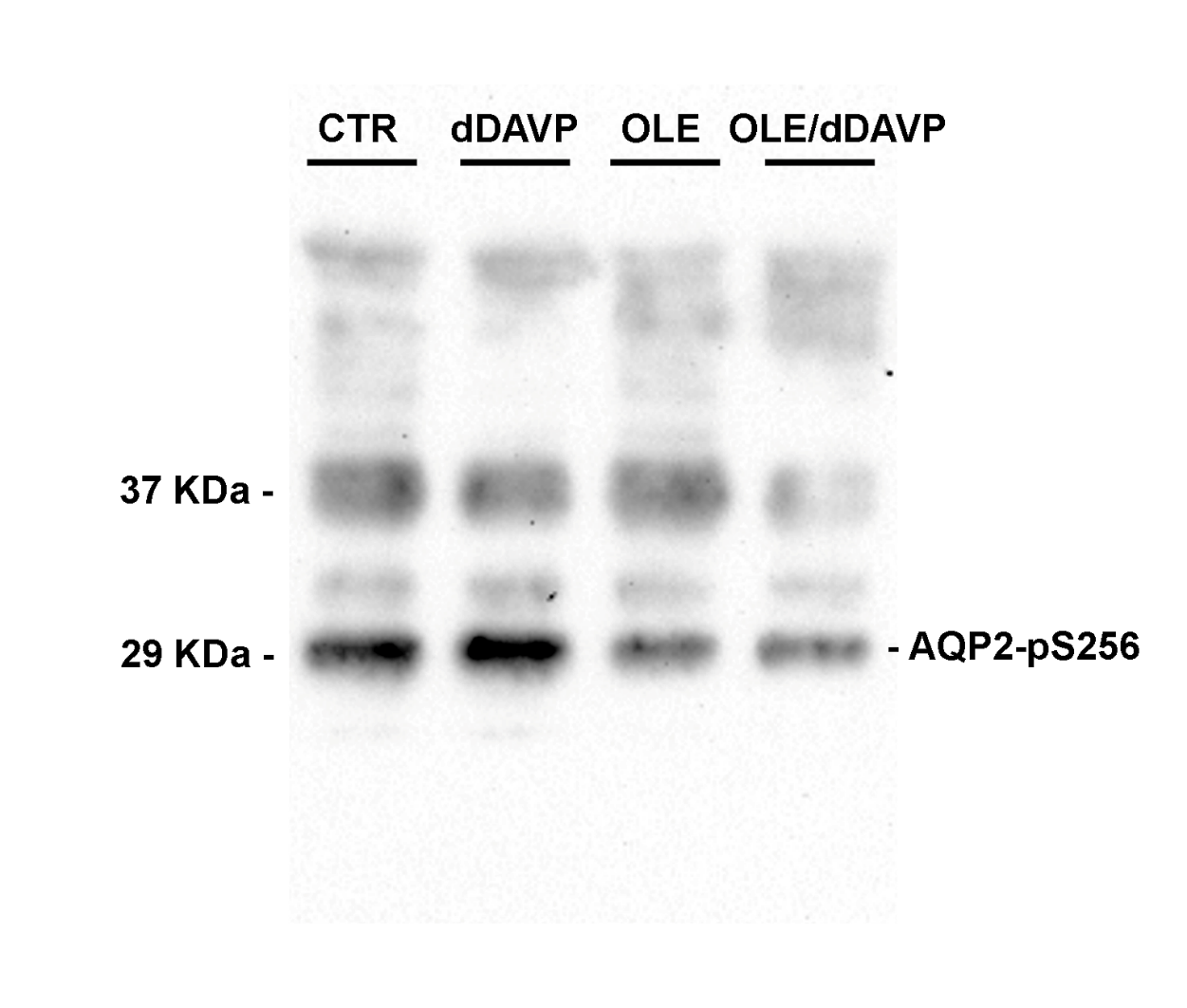


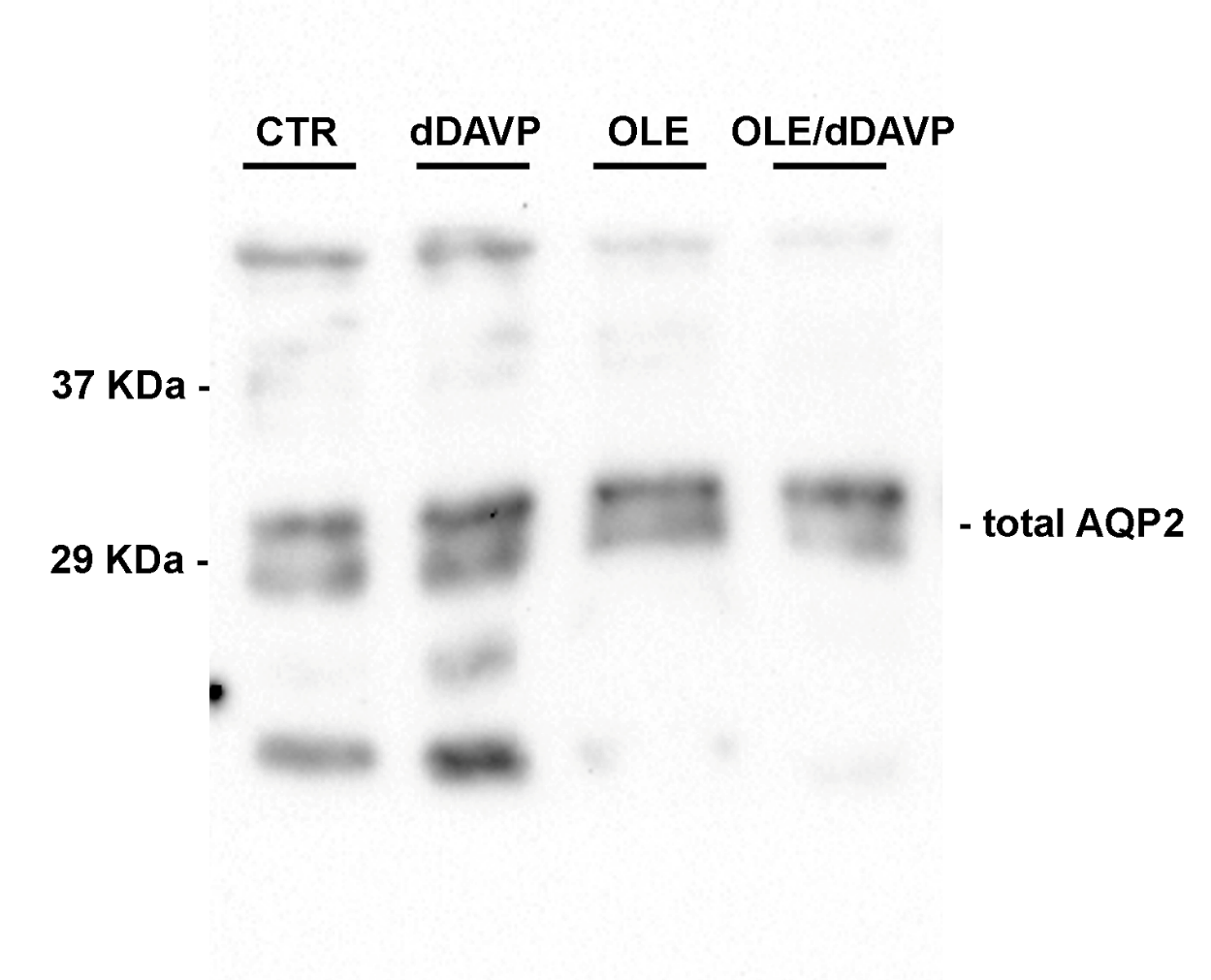


**Fig. 2B**


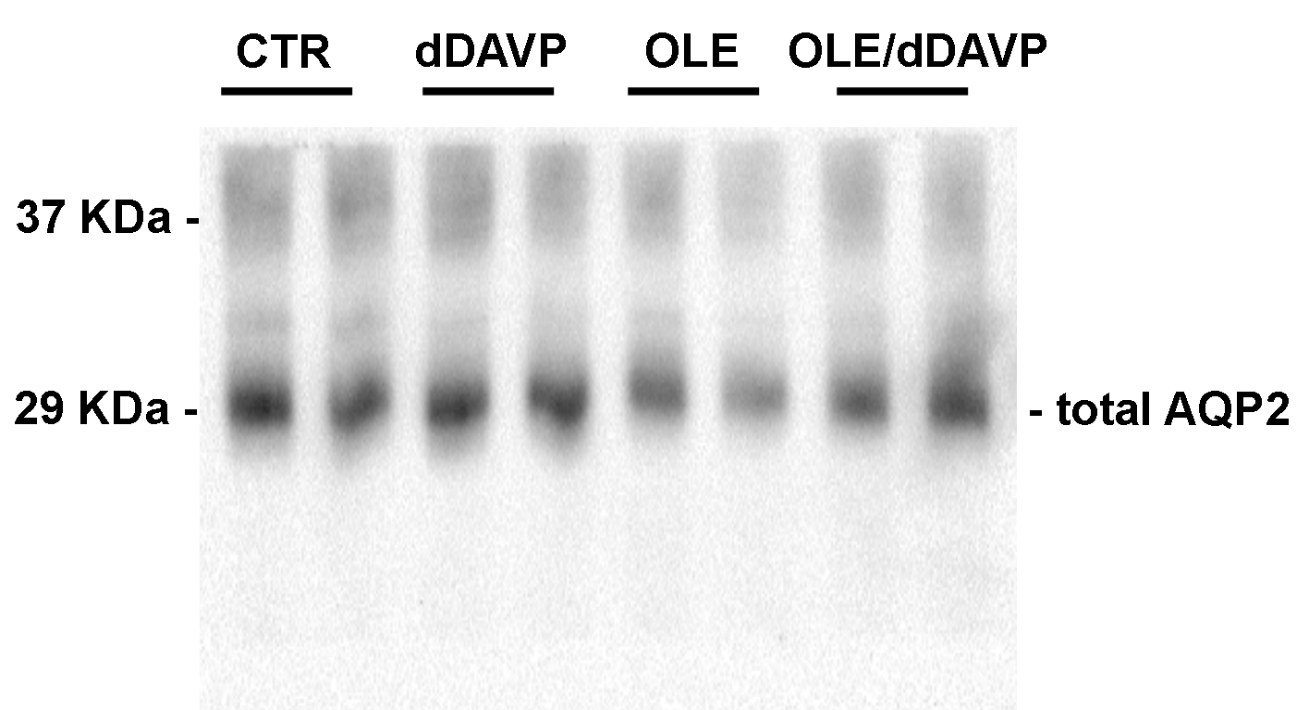


**Fig. 8B**


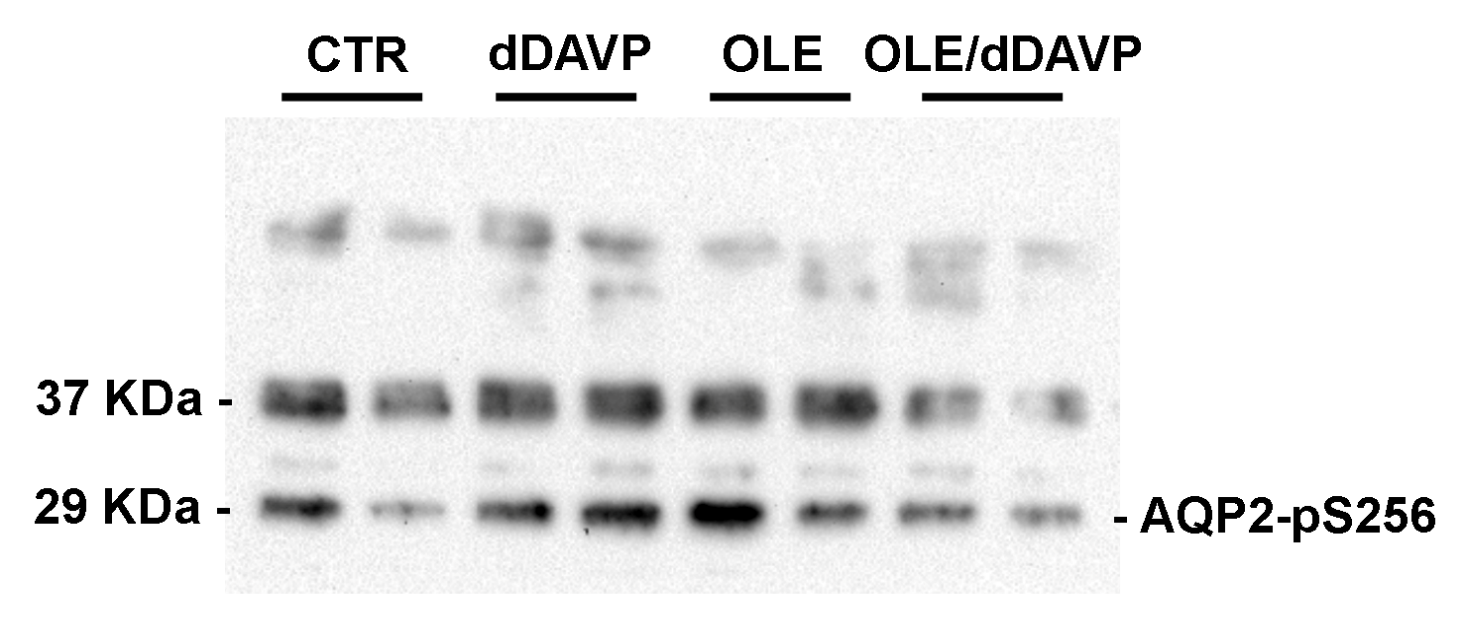


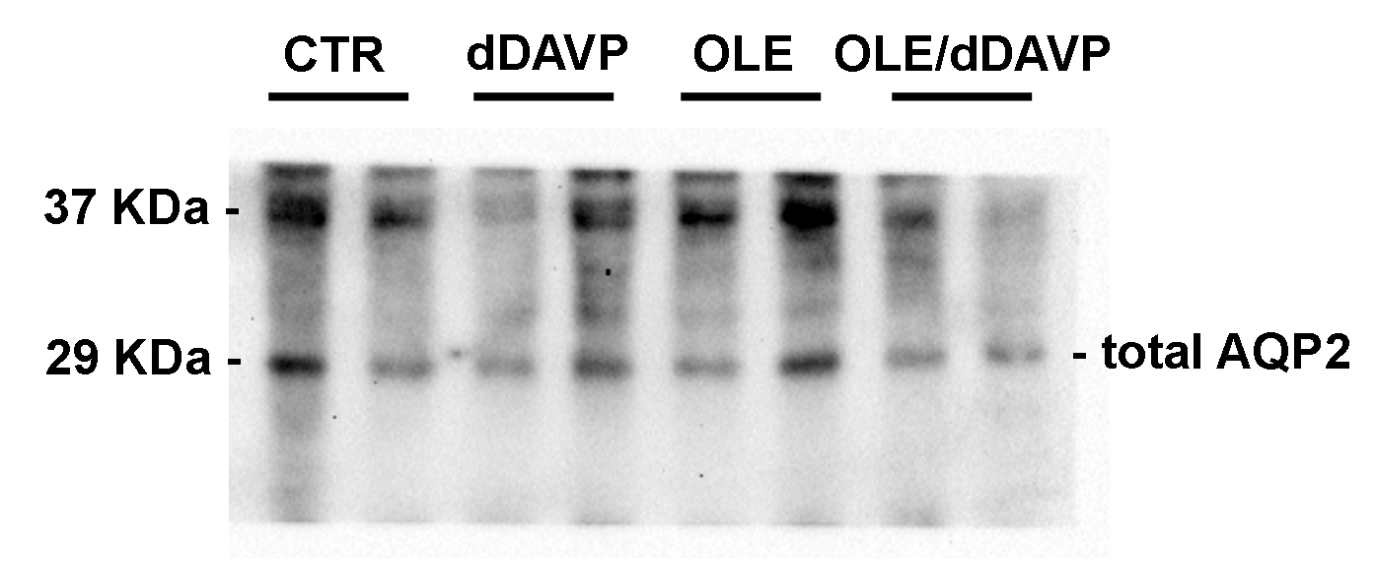


**Fig. 9A**
